# Upper Gastrointestinal Bleeding Revealing Renogastric Fistula: A Case Report and Literature Review

**DOI:** 10.1155/2020/3018347

**Published:** 2020-03-19

**Authors:** Yassine Mellagui, Inass Haouli, Jamal Ouachaou, Mohammed Aabdi, Houssam Bkiyar, Brahim Housni

**Affiliations:** Intensive Care Unit, Mohammed VI University Hospital Center, Faculty of Medicine and Pharmacy of Oujda, Mohammed VI University, Oujda, Morocco

## Abstract

A renogastric fistula is a rare complication defined by abnormal communication between the kidney and the stomach. We report the case of a patient admitted to the intensive care unit for hemorrhagic shock following upper gastrointestinal bleeding whose radiological investigations revealed a fistulated renal hematoma in the stomach.

## 1. Introduction

A renogastric fistula is an extremely rare complication, always affecting the left kidney, and it is among the rarest fistulas between the urinary and digestive tracts [1].

## 2. Case Report

A 53-year-old patient followed for chronic renal failure (whose etiology is bilateral renal polycystosis in the hemodialysis stage) and also followed for bladder tumor (urothelium grade 2) was admitted to the intensive care unit for the management of hemorrhagic shock following upper digestive bleeding.

The clinical examination found the patient with GSC 13/15, with arterial hypotension at 85/46 mmHg, tachycardic at 115, with diuresis at 0.3 ml/kg/h, and with macroscopic hematuria. The respiratory examination found the patient polypneic at 26 and with ambient air saturation at 85%. The abdominal examination found an epigastric sensitivity with touch and rectal absence of melena.

The biological assessment had objectified hemoglobin level at 5.9 g/dl, platelets at 195,000/mm^3^, TP at 60%, renal failure with creatinemia at 85 mg/l and urea level at 1.1 g/l, hyponatremia at 132 mEq/l, and hyperkalemia at 5.9 mEq/l.

Our management was to transfuse the patient with 2 units of red blood cells, administer norepinephrine on a central venous catheter, and make hypokalemic measurements.

**Figure 1:**
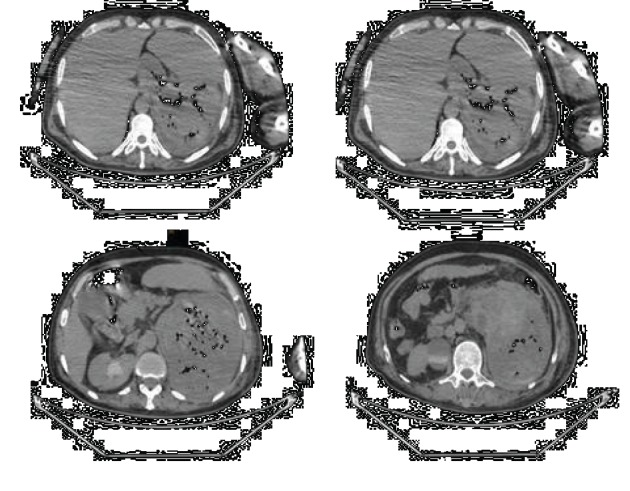
Abdominal scanner without contrast injection showing a large hematoma of the left kidney fistulated in the stomach.

After stabilization, the patient had an abdominal CT scan that objectified a large hematoma of the left kidney fistulated in the stomach (Figure 1).

Unfortunately, the patient died after the second episode of haematemesis of great abundance and complicated refractory hemorrhagic shock, before a hemostasis procedure was performed.

## 3. Discussion

A fistula is an abnormal communication between 2 epithelium-lined cavities. Urointestinal fistulas can occur between any part of the urinary system and the digestive tract. They are rare and renogastric fistulas are the least common fistula between the urinary and digestive tracts. They are most often of chronic inflammatory origin, secondary to kidney stones or traumatic origin [2, 3]. Normally, if a fistula should develop between the kidney and the gastrointestinal tract, it will do so at sites directly related the kidney: either between the right kidney and the duodenum or the kidney and the colon [4]. The most frequent clinical signs found in cases of urointestinal fistulas are nausea-vomiting, lumbar pain, hematuria, pyuria, and fever [5]. Diagnosis is based on CT scan, which determines the nature, location, and sometimes the etiology and complications of these fistulas [6]. The treatment consists in majority of cases in nephrectomy and digestive suture [7].

## 4. Conclusion

A renogastric fistula is a rare complication with a variety of etiologies and clinical signs, and diagnosis is based on CT scan and treatment is often surgical.
